# Clinical outcomes of tibial cortex transverse transport versus antibiotic-loaded bone cement for Wagner grade 3–4 diabetic foot ulcers: a real-world retrospective cohort study

**DOI:** 10.3389/fendo.2026.1838948

**Published:** 2026-06-03

**Authors:** Muhan Li, Chenghao Zhu, Zhongxian Xu, Xiwen Feng, Guangming Zheng, Yongjun Li

**Affiliations:** 1 Department of Foot and Ankle Surgery, ShunDe Hospital of Guangzhou University of Chinese Medicine, Foshan, Guangdong, China; 2 Department of Traumatic Orthopedics, ShunDe Hospital of Guangzhou University of Chinese Medicine, Foshan, Guangdong, China

**Keywords:** ankle-brachial index, antibiotic-loaded bone cement, diabetic foot ulcers, limb salvage, major amputation, tibial cortex transverse transport, unplanned return to the operating room, Wagner grade

## Abstract

**Background:**

Diabetic foot ulcers (DFUs) are a primary cause of non-traumatic lower-extremity amputations. Wagner grade 3–4 ulcers, which are often complicated by deep infection and impaired perfusion, present significant management challenges. Antibiotic-loaded bone cement (ALBC) establishes a localized high-concentration antimicrobial environment and facilitates dead-space management, while tibial cortex transverse transport (TTT) is designed to enhance pedal perfusion via distraction-induced angiogenesis, thereby promoting wound healing. Rather than being interchangeable procedures, ALBC and TTT are two distinct, clinically selected surgical limb-salvage approaches. ALBC primarily addresses infection control and dead-space management, while TTT targets perfusion improvement. However, there is a notable lack of direct comparative evidence between these two approaches.

**Methods and analysis:**

A retrospective analysis of clinical data was conducted on patients with Wagner grade 3–4 DFUs who received TTT or ALBC at our center from January 2020 to December 2023. Following standardized initial management and multidisciplinary reassessment, patients were assigned to either the TTT or ALBC pathway, based on their dominant clinical problem, infection burden, perfusion status, and vascular anatomical feasibility. We collected and analyzed perioperative indicators and postoperative outcomes, with further stratification by Wagner grade for a comprehensive evaluation. Kaplan–Meier analysis was used to evaluate major amputation-free survival. Factors associated with the postoperative 3-month ankle–brachial index (ABI) were assessed using multivariable linear regression, while multivariable binary logistic regression identified independent factors associated with unplanned return to the operating room (URTOR).

**Results:**

There were no significant differences between the groups regarding the percentage area reduction (PAR) at 3 months or in the rates of major amputation. Kaplan–Meier analysis revealed no statistically significant difference in major amputation-free survival between the TTT and ALBC groups. However, stratified analyses revealed marked findings for specific patient categories. Among patients with Wagner grade 4 DFUs, the TTT group demonstrated a significantly higher PAR at 6 months compared to the ALBC group (P = 0.002) and a lower rate of unplanned return to the operating room (URTOR) (P = 0.018). Similarly, for patients with Wagner grade 3 DFUs, the TTT group showed a significantly lower URTOR rate (P = 0.012). Furthermore, the TTT group demonstrated a significantly greater improvement in the ankle–brachial index (ABI) (P < 0.05), while the ALBC group had a notably shorter time to infection clearance (P < 0.001). In multivariable analyses, treatment group and preoperative ABI were significantly associated with postoperative 3-month ABI. Treatment with TTT was independently associated with lower odds of URTOR compared with ALBC, and higher preoperative ABI was associated with reduced URTOR risk.

**Conclusion:**

ALBC and TTT represent two clinically selected surgical limb-salvage strategies with different therapeutic priorities for Wagner grade 3–4 DFUs complicated by coexisting infection and perfusion impairment. ALBC may be more appropriate when infection control, osteomyelitis, abscess formation, or dead-space management is the principal clinical concern, whereas TTT may be more suitable when impaired distal perfusion is the dominant factor limiting wound healing. In this real-world cohort, TTT was associated with greater improvement in perfusion and a lower rate of unplanned reintervention, particularly in patients with Wagner grade 4 ulcers; meanwhile, ALBC was associated with faster infection clearance. Collectively, these findings suggest an individualized treatment approach following standardized initial management, where treatment selection is determined by ulcer severity, infection burden, perfusion status, and vascular anatomy.

## Introduction

Diabetic foot ulcers (DFUs) are a prevalent and severely disabling chronic complication of diabetes. They are also one of the primary risk factors for non-traumatic lower-extremity amputation (2). The pathogenesis of DFUs is multifactorial, with peripheral neuropathy and lower-extremity vascular disease frequently interacting synergistically, resulting in unrecognized foot injuries and diminished reparative capacity. Notably, once secondary infection occurs, tissue destruction can escalate rapidly, thereby extending the duration necessary for wound bed preparation and healing (2).

Current consensus statements and international guidelines underscore multidisciplinary management of DFUs, including debridement-based wound care, off-loading, and systemic infection control, and perfusion assessment. For patients with impaired perfusion, the potential benefit of revascularization need to be evaluated, and revascularization be conducted where clinically necessary (3, 4). However, in clinical practice, wound healing is often unpredictable. Wagner grade 3–4 DFUs are frequently accompanied by deep infection, tissue loss, and varying degrees of perfusion impairment. Even when standard treatment pathways are followed, outcomes may be unfavorable, resulting in delayed healing and poor limb salvage. The above data show that antibiotic-loaded bone cement (ALBC) and tibial transverse transport (TTT) are two alternative surgical strategies that are potentially effective in selected patients with complex DFUs. ALBC is a commonly used adjunctive surgical strategy for local infection control and dead-space management in diabetic foot ulcers (1), providing sustained high local antibiotic concentrations at the lesion site, even under conditions of impaired perfusion and limited antibiotic penetration, while reducing the risk of systemic toxicity (5). In contrast, TTT is based on the Ilizarov tension–stress principle, whereby cyclic distraction of a tibial cortical segment promotes neovascularization and stimulates pedal microcirculation, thus establishing a more stable perfusion foundation for wound healing (6). TTT is most commonly utilized for DFU patients exhibiting ischemic manifestations, particularly when their healing progress remains sluggish despite comprehensive conventional management (7). Notably, current international guidelines for the management of DFUs complicated by peripheral arterial disease primarily aims to perform perfusion assessment, risk stratification, and revascularization when indicated (4), whereas in Chinese clinical practice, TTT, as a locally developed limb-salvage technique, has been included into relevant domestic expert consensus and is employed in some centers to treat complex and refractory DFUs (8). Moreover, in specific clinical settings in China, for selected patients with Wagner grade 3–4 DFUs presenting with both infection and perfusion impairment, ALBC and TTT represent mechanistically distinct but clinically complementary limb-salvage strategies, targeting infection control and perfusion impairment, respectively. In certain patients with coexisting infection and impaired perfusion, these approaches may be considered alternative or sequential options depending on the dominant pathological process after standardized initial management. Although both strategies are applied in such patients, direct comparative evidence regarding their relative efficacy and safety remains limited. Consequently, we conducted a retrospective cohort comparison of these two strategies and, recognizing the heterogeneity in disease severity between Wagner grades 3 and 4, implemented stratified analyses in addition to the overall assessment.

## Methods

### Study design and setting

**Figure 1:**
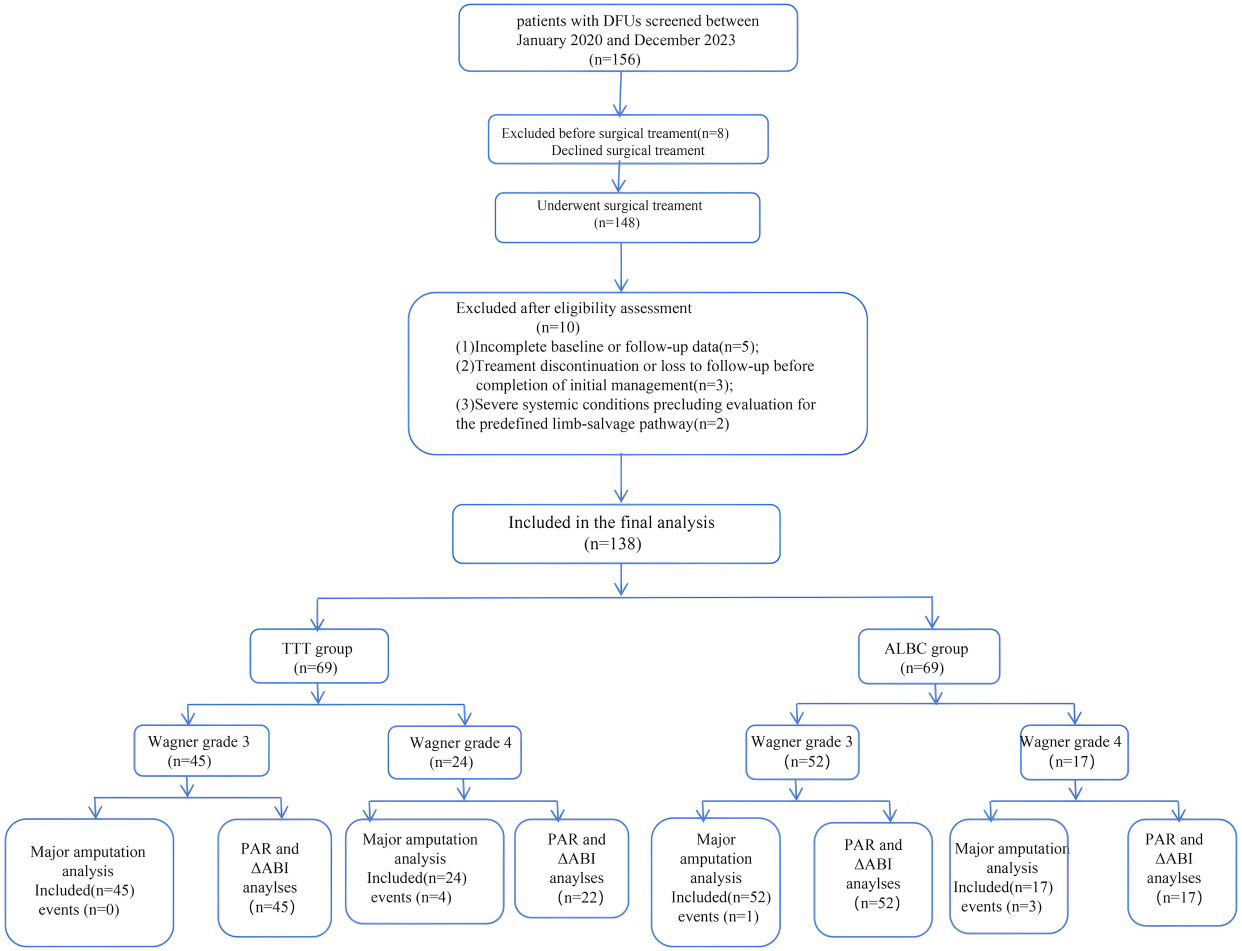
Flow chart of patient screening and final study population.

This retrospective cohort study enrolled 156 patients with diabetic foot ulcers between January 2020 and December 2023. Among them, 8 declined surgical treatment and were excluded. The remaining 148 patients underwent surgical treatment and were further evaluated for eligibility. Ten patients were further excluded: 5 had incomplete baseline or follow-up data, 3 discontinued treatment or were lost to follow-up before completion of initial management, and 2 had severe systemic conditions precluding evaluation within the predefined limb-salvage pathway. Finally, 138 patients were included in the final analysis: 69 in the TTT group and 69 in the ALBC group. The patient screening and study selection process is presented in **Figure 1**.

### Patients

The selection of patients was based on the following criteria: (1): a clinical diagnosis of type 1 or type 2 diabetes mellitus; (2); a Wagner grade 3 or 4 diabetic foot ulcer; (3); receipt of standardized initial management after admission; (4) completion of multidisciplinary reassessment at the predefined decision time point (48–72 hours after initial debridement), and then allocated to either the TTT or ALBC pathway; in this context, patients were considered to have a “refractory” clinical course when they did not demonstrate satisfactory improvement after standardized initial management and required escalation to surgical limb-salvage strategies based on multidisciplinary assessment; and (5) availability of complete baseline and follow-up outcome data for analysis. A history of previous lower-extremity revascularization before admission was not itself an exclusion criterion. Patients with such a history could be included if multidisciplinary vascular reassessment after admission did not indicate a need for priority repeat revascularization before allocation to the predefined local limb-salvage pathway. The following exclusion criteria were applied: (1) substantial missing baseline or follow-up data; (2) discontinuation of treatment or loss to follow-up before completion of standardized initial management; (3) CTA after admission showing major proximal arterial occlusion, including superficial femoral artery or popliteal artery occlusion, or absence of effective distal arterial runoff to the lower leg or ankle, indicating the need for priority vascular or interventional evaluation for possible revascularization before consideration of the predefined local limb-salvage pathway; or (4) severe systemic conditions that make it difficult to evaluate within the predefined limb-salvage pathway, including septic shock or acute cerebrovascular or cardiovascular events.

### Standardized initial management and pathway allocation

All patients received standardized initial management following admission, which included primary debridement, collection of deep tissue or wound specimens for microbiological testing and wound exudate culture, empirical or targeted systemic antimicrobial therapy, lower-extremity vascular assessment (CTA), wound and infection assessment, glycemic management, and off-loading. Lower-extremity vascular assessment was performed using ABI and CTA to evaluate perfusion status, proximal arterial patency, and distal arterial runoff.

The multidisciplinary reassessment performed 48–72 hours after initial debridement was defined as the treatment pathway allocation time point. At this predefined decision point, wound severity, ischemia severity, and foot infection severity were graded using the WIfI classification framework (9). Perfusion status was evaluated using preoperative ABI and CTA-based assessment of distal arterial runoff, while infection-related characteristics, including abscess or deep soft tissue infection, osteomyelitis, and post-debridement dead-space management requirements, were also documented (10, 11). Regarding treatment allocation, patients were assigned to different pathways based on the results of clinical reassessment, guided by the dominant pathological process, as determined by the WIfI classification, infection-related variables, and CTA-based assessment of distal arterial runoff, together with anatomical feasibility.

The indication for TTT was determined by multidisciplinary clinical judgment and by reference to the Chinese expert consensus on tibial cortex transverse transport for diabetic foot ulcers (8). In the present study, perfusion impairment was assessed using preoperative ABI and CTA-based evaluation of distal arterial runoff. According to the expert consensus, TTT is considered suitable for selected patients with severe DFUs who have patency of the superficial femoral artery and popliteal artery, and at least one of the anterior tibial, posterior tibial, or peroneal arteries reaching the ankle level. Therefore, patients with major proximal arterial occlusion, including superficial femoral artery or popliteal artery occlusion, or absence of effective distal arterial runoff to the lower leg or ankle, were not included in the predefined TTT versus ALBC comparative cohort. Such patients were considered to require priority vascular or interventional evaluation for possible revascularization before consideration of local limb-salvage procedures. Accordingly, TTT was not used as a substitute for conventional revascularization or as a no-option ischemia therapy, but as a perfusion-oriented adjunctive limb-salvage procedure in selected patients with preserved proximal arterial patency and residual distal arterial runoff.

Patients with coexisting infection and impaired perfusion who met the above vascular criteria were considered for the TTT pathway, particularly when inadequate distal perfusion was determined to be the major limiting factor for wound healing and when no deep infection, abscess formation, or significant post-debridement dead space requiring priority management was present (12).

The ALBC pathway was selected for patients who persistently showed obvious local signs of infection at reassessment despite standardized initial management, particularly when deep infection, osteomyelitis, abscess formation, or substantial post-debridement dead-space management requirements were detected. Patients were preferentially assigned to the ALBC pathway when control of deep infection and dead-space management was the principal clinical concern (13).

**Figure 2:**
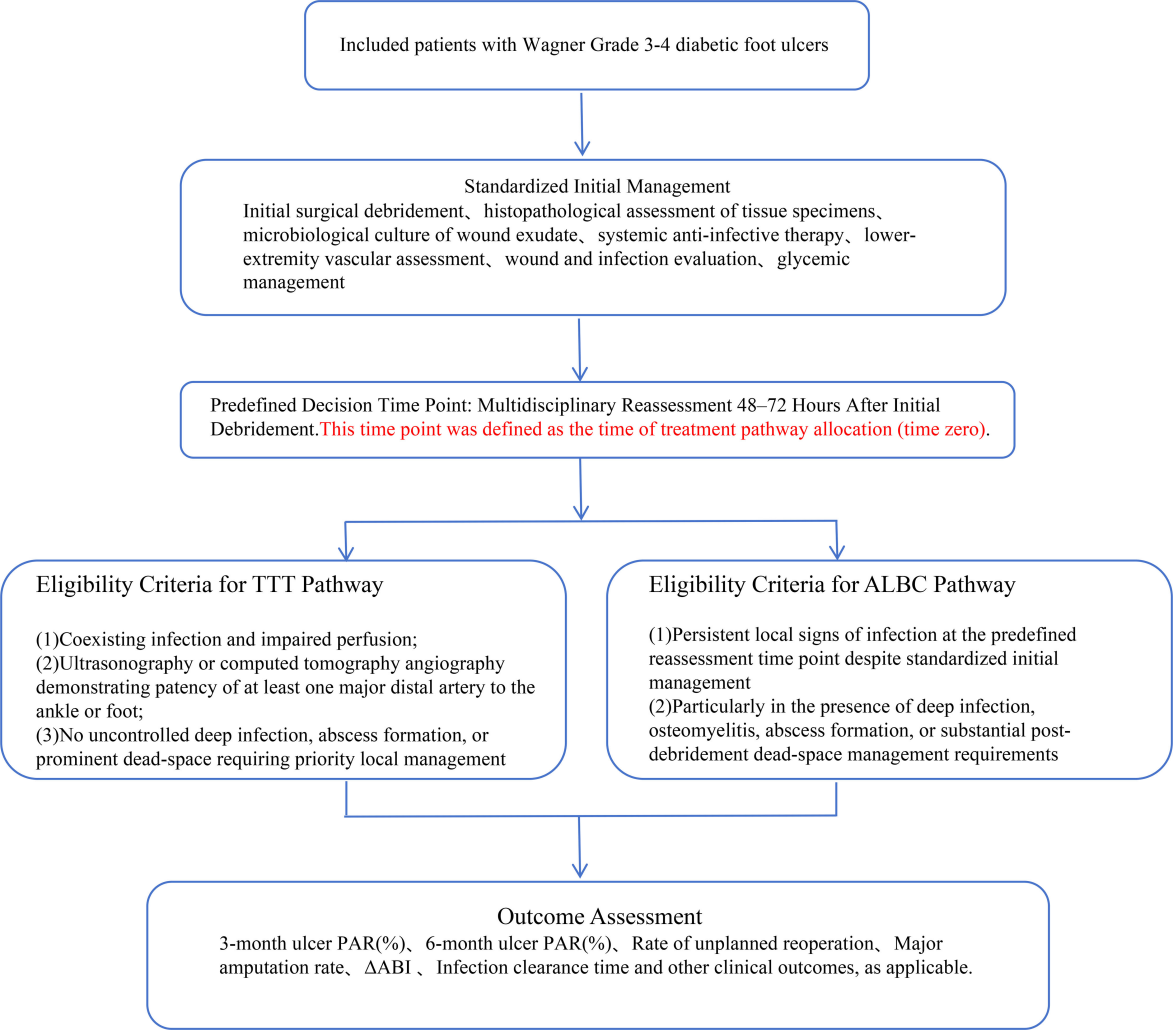
Clinical pathway allocation in patients with Wagner grade 3–4 diabetic foot ulcers.

The treatment allocation process is shown in **Figure 2**. All procedures in both groups were performed by the same surgical team.

### ALBC procedure

Under epidural anesthesia or combined spinal–epidural anesthesia, the incision was planned based on the extent of infection. Thorough debridement was performed, including the excision of necrotic soft tissue. For cases with deep involvement, debridement was extended as necessary. In instances of bony involvement, infected bone was removed until a fresh, bleeding bony surface was reached. The wound was then irrigated thoroughly with hydrogen peroxide, diluted povidone–iodine, and 0.9% saline, and hemostasis was achieved.

**Figure 3:**
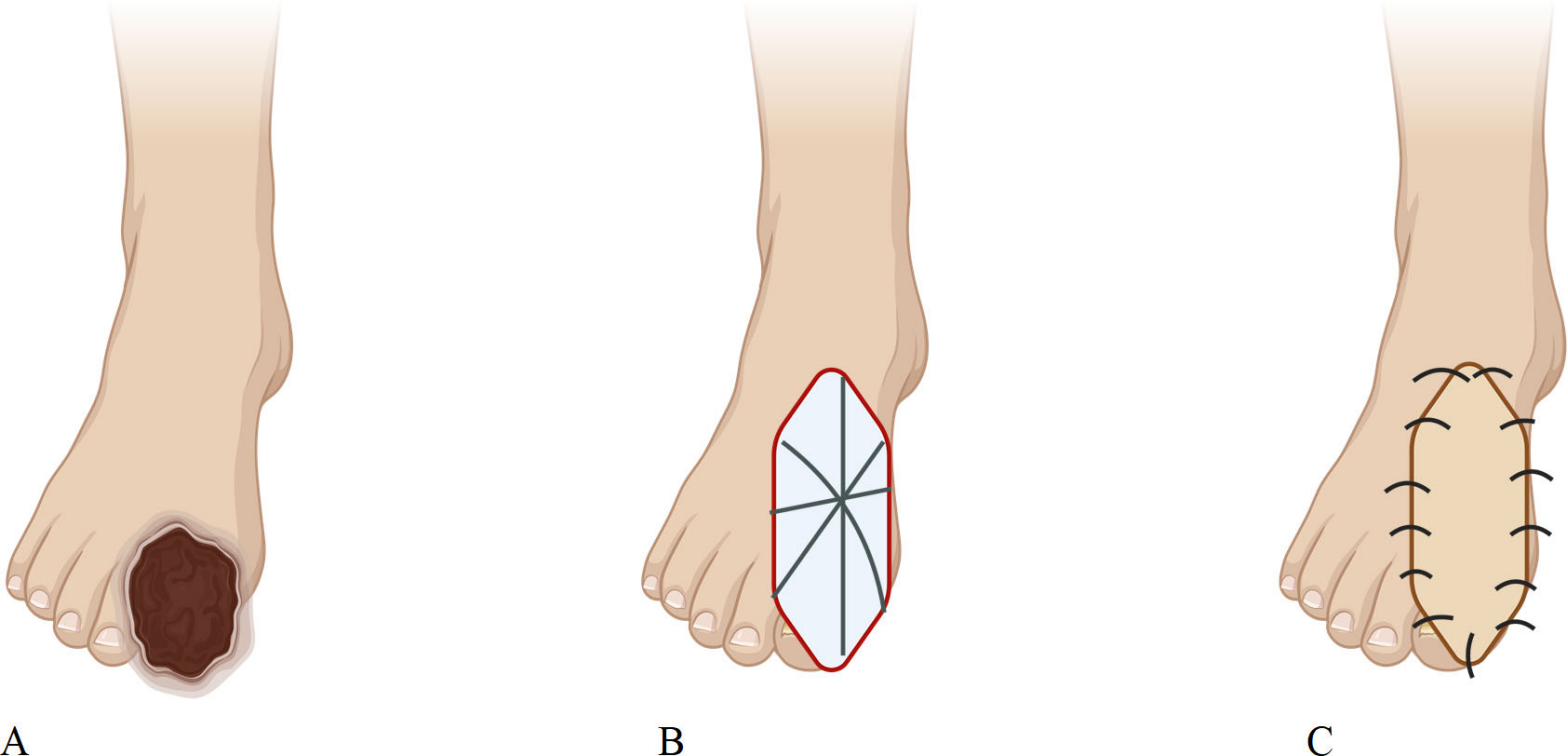
Flow chart of ALBC procedure. **(A)** Wound assessment and debridement. **(B)** Wound-edge marking and closure planning. **(C)** Layered closure: interrupted deep tension-relieving sutures first, followed by subcutaneous and skin closure, ensuring well-perfused edges and uniform tension.

Antibiotic-loaded bone cement was prepared at a fixed ratio (e.g., 40 g of bone cement mixed with 2 g of vancomycin) and implanted in pieces, leaving deliberate gaps to facilitate drainage (14). The wound was then covered with vacuum sealing drainage (VSD). Approximately 20 days postoperatively, infection control and granulation tissue formation were reassessed. If adequate, the bone cement was removed, and repeat debridement was performed, followed by elective definitive closure using direct suturing, skin grafting, or flap coverage. If the assessment indicated inadequate progress, repeat debridement and bone cement exchange were performed until the criteria for secondary closure were met (**Figure 3**).

### TTT procedure

Under epidural anesthesia or combined spinal–epidural anesthesia, two longitudinal incisions of approximately 3 cm were made on the anteromedial aspect of the proximal tibia, located 3–5 cm distal to the tibial tuberosity. After exposing the periosteum, a cortical bone window measuring approximately 5.0 × 1.5 cm was created on the medial tibial cortex. Multiple drill holes were then made, followed by corticotomy to form a mobile cortical segment. Subsequently, a movable half-pin (approximately 3.0 mm, unicortical) and a fixed half-pin (approximately 4.0 mm, bicortical) were inserted, and the external fixator was assembled. For patients with concomitant infection or necrosis, debridement was performed concurrently, with VSD applied when indicated.

**Figure 4:**
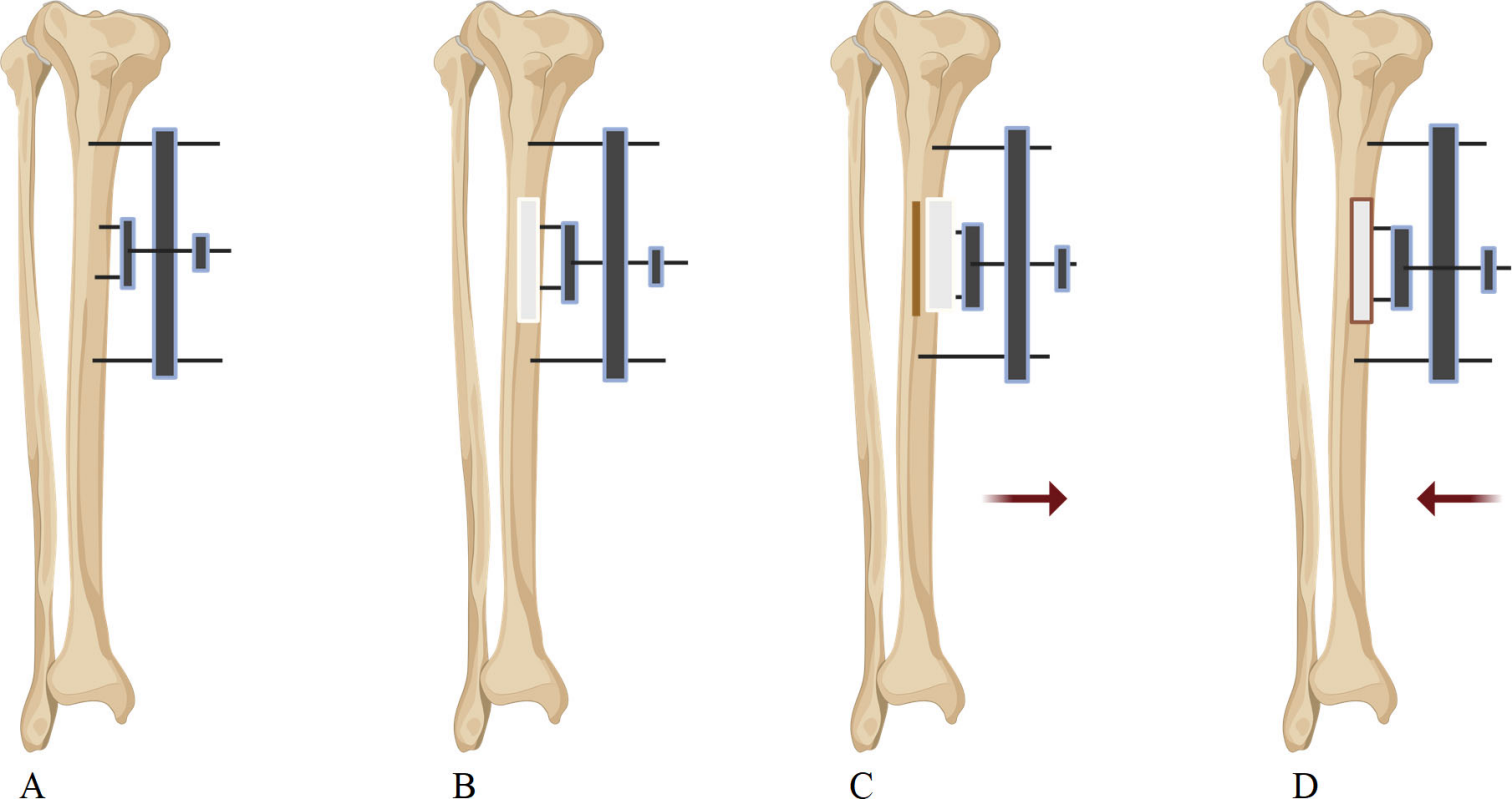
Flow chart of TTT procedure. **(A)** External fixator assembly and half-pin placement. **(B)** Corticotomy creating an approximately 5 × 1.5 cm rectangular cortical bone window. **(C)** Transverse transport initiated on postoperative day 5 (14 days; 0.5 mm per session, twice daily). **(D)** After a 3-day dwell period, the segment was transported back to its original position (14 days). The external fixator was removed 2 weeks later.

Beginning on postoperative day 5, the cortical segment was transported laterally at a rate of 1 mm/day for 14 days. It was then kept in the distracted position for an additional 3 days before being transported back to its original position at the same rate for another 14 days. The external fixator was maintained for 6–8 weeks and was removed only after radiographic confirmation of bone-window healing (**Figure 4**).

### Concomitant background therapy

Following pathway allocation, patients in both groups received standardized background care, that included wound dressing changes and local care, continued systemic antimicrobial management as required, glycemic management, and postoperative off-loading with loose customized footwear.

### Follow-up time windows and assessment procedures

Here, we retrospectively collected preoperative baseline data and postoperative follow-up information, with a follow-up observation period of 12 months after surgery. Baseline characteristics, including wound grade, ischemia grade, foot infection grade, and relevant infection-related parameters, were extracted from medical records generated during the multidisciplinary pre-treatment assessment, conducted after standardized initial management and immediately prior to treatment-pathway allocation. Outcome assessment was conducted through routine clinical follow-up visits and documentation from electronic medical records. In addition, lower-extremity revascularization status was extracted from medical records and follow-up documentation, including previous revascularization before admission and additional revascularization during follow-up after the TTT or ALBC procedure. Data on wound assessments were extracted from the 3-month and 6-month postoperative follow-up periods. The last valid follow-up record, obtained at least 12 months after surgery, was utilized to evaluate the primary outcome; patients with follow-up durations shorter than 12 months were excluded from this analysis. Wound assessments during each time window included measurements of ulcer area and depth, infection status, exudate levels, and granulation tissue formation. To minimize observer bias, wound morphology was independently reviewed and documented by researchers not involved in the surgical procedures, relying on a retrospective review of medical records and imaging data.

### Wound area measurement

Ulcer area was measured using digital planimetry with ImageJ. A disposable sterile ruler was placed next to the wound as a scale, ensuring sterile conditions. Wound photographs were taken with a standard camera at a consistent shooting distance and angle, while keeping the camera axis as perpendicular to the wound plane as possible to minimize projection error. The images were then imported into ImageJ for scale calibration, tracing of the wound edges, and calculation of the area (15).

### Outcomes and definitions

The primary outcome was major amputation-free survival, defined as the interval from the date of the index surgery to the occurrence of major amputation, with patients censored at the date of the last valid follow-up if no major amputation had occurred. Major amputation was defined as lower-extremity amputation proximal to the ankle (16). Secondary outcomes included the percentage area reduction (PAR) of ulcers, measured within the 3-month and 6-month postoperative assessment windows. PAR was determined as the difference between the baseline ulcer area and the follow-up ulcer area, divided by the baseline ulcer area and multiplied by 100%.

Unplanned return to the operating room (URTOR) within 6 months postoperatively was defined as any unplanned reoperation/reintervention following the index surgery due to complications, inadequate infection control, wound deterioration, device-related problems, perfusion deterioration, or treatment strategy adjustment. Any unplanned lower-extremity revascularization meeting this definition was counted as a URTOR event, whereas revascularization performed later during follow-up was recorded descriptively and was not included in the URTOR endpoint. Protocol-specified staged procedures, including planned removal of the external fixator and preplanned elective wound coverage in the TTT group, as well as planned bone cement removal and staged closure in the ALBC group, were not counted as URTOR events (17–19). To improve interpretability, URTOR events were categorized according to the first URTOR event experienced by each patient into four groups: unplanned fixation/device revision, unplanned debridement/drainage, non-amputation salvage reoperation/reintervention, and minor distal amputation below the ankle. As a heterogeneous intermediate composite endpoint, URTOR was used to reflect early postoperative treatment instability, unplanned surgical escalation, or the need for additional surgical optimization. Therefore, it was considered complementary to, rather than a substitute for, more definitive outcomes such as complete wound healing, major amputation, or major amputation-free survival. Time to infection clearance was defined as the duration from the date of the initial surgical intervention to the first documented evidence of infection control. Infection control was evaluated considering the resolution of clinical signs of infection, as well as the conversion of deep wound tissue cultures from positive to negative results (20). ABI was measured before surgery and reassessed at 3 months postoperatively. ΔABI was defined as the difference between ABI at 3 months postoperatively and preoperative ABI (21). Hospital length of stay (LOS) was calculated as the period from the day of admission to the day of discharge, specifically for hospitalizations associated with the diagnosis and treatment of DFU (22).

### Statistical analysis

Statistical analyses were conducted utilizing IBM SPSS Statistics (version 27.0). Following an assessment of normality, continuous variables exhibiting a normal distribution are presented as mean ± standard deviation (SD) and were compared between groups using the independent-samples t-test. Continuous variables not conforming to a normal distribution are reported as median (interquartile range, IQR) and were compared utilizing the Mann–Whitney U test. Categorical variables are expressed as number (percentage) and were compared using the Pearson chi-square test; when the expected frequency in any cell of a 2×2 contingency table was < 5, Fisher’s exact test (two-sided) was employed. For categorical comparisons involving tables larger than 2×2, the Fisher-Freeman-Halton exact test (two-sided) was applied. Within-group comparisons across different time points were performed using the paired t test or the Wilcoxon signed-rank test, as deemed appropriate. Major amputation-free survival was analyzed as a time-to-event outcome using the Kaplan–Meier method, and differences between groups were assessed using the log-rank test. Patients who underwent major amputation were considered to have reached the endpoint at the time of the event and were no longer included in subsequent wound-healing or perfusion-related outcome analyses. To further evaluate factors associated with postoperative vascular recovery, multivariable linear regression analysis was performed with postoperative 3-month ABI as the dependent variable. The independent variables entered into the model included Wagner grade, treatment group, osteomyelitis, abscess or deep soft tissue infection, and preoperative ABI. Regression coefficients (B), standard errors (SE), standardized β coefficients, t values, and P values were reported. To identify factors independently associated with URTOR, multivariable binary logistic regression analysis was performed. URTOR was entered as the dependent variable, and Wagner grade, treatment group, osteomyelitis, abscess or deep soft tissue infection, and preoperative ABI were included as independent variables. Odds ratios (ORs) with 95% confidence intervals (CIs) were calculated. In the logistic regression model, preoperative ABI was multiplied by 10, so the OR represents the change in odds of URTOR for each 0.1-unit increase in preoperative ABI. All statistical tests were two-sided, with a significance threshold set at P < 0.05.

## Results

### Baseline characteristics

Overall, 138 patients presenting with Wagner grade 3–4 DFUs were included in the final analysis (with 69 per group). Among them, 97 patients developed Wagner grade 3 ulcers, with 45 patients in the TTT group and 52 in the ALBC group; 41 patients had Wagner grade 4 ulcers, with 24 patients in the TTT group and 17 in the ALBC group. General demographic and baseline clinical characteristics were comparable between the TTT and ALBC groups (**Table 1**). To further characterize the patients’ status at the treatment-decision point, wound-, ischemia-, and infection-related parameters after standardized initial management and immediately prior to treatment-pathway allocation are summarized in **Supplementary Table 1**. Wound grade distribution was similar between groups (P = 0.634). Ischemia grade distribution showed no statistically significant difference (P = 0.059), whereas the number of patent distal runoff vessels on CTA differed significantly, with a higher proportion of one-vessel runoff in the TTT group and higher proportions of two- or three-vessel runoff in the ALBC group (P < 0.001). Infection-related characteristics also differed substantially: the ALBC group had a higher foot infection grade distribution, as well as higher proportions of abscess or deep soft tissue infection, osteomyelitis, and requirement for dead space management (all P < 0.001) (**Supplementary Table 1**).

**Table 1:** The baseline characteristics of patients undergoing TTT or ALBC.

Characteristics	TTT group (n=69)	ALBC group (n=69)	P value
Age (years)	69.75 ± 9.83	66.09 ± 14.46	0.084
Body Mass Index (kg/m²)	23.07 ± 3.16	23.34 ± 3.60	0.641
Sex, n (%)			0.393
Female	29 (42.03)	34 (49.28)	
Male	40 (57.97)	35 (50.72)	
Wagner grade, n (%)			0.192
III	45 (65.22)	52 (75.36)	
IV	24 (34.78)	17 (24.64)	
Ulcer area (cm²)	10.000 (4.8, 30.0)	9.000 (5.0, 28.0)	0.925
Diabetes duration, n (%)			0.394
≤ 5 years	30 (43.48)	35 (50.72)	
> 5 years	39 (56.52)	34 (49.28)	
Preoperative HbA1c (%)	7.59 ± 2.15	7.59 ± 1.97	0.980
Ulcer microbiology, n (%)			0.407
Culture-negative	9 (13.04)	9 (13.04)	
Monomicrobial infection	29 (42.03)	32 (46.38)	
Polymicrobial infection	31 (44.93)	28 (40.59)	
ABI pre	0.49 ± 0.17	0.54 ± 0.15	0.070
Preoperative WBC (×10^9^/L)	9.740 (7.3, 11.4)	8.480 (6.9, 13.3)	0.313
Preoperative CRP (mg/L)	14.880 (3.1, 66.7)	19.640 (2.2, 93.8)	0.452
Preoperative albumin (g/L)	35.86 ± 5.24	36.69 ± 4.70	0.329

HbA1c, glycated hemoglobin; WBC, white blood cell count.

### Revascularization status

Revascularization status was summarized according to its timing relative to admission, treatment-pathway allocation, and postoperative follow-up. Before admission, ten patients had previously undergone lower-extremity revascularization, including four patients in the TTT group and six patients in the ALBC group. After admission, all patients underwent multidisciplinary vascular assessment before treatment-pathway allocation. No patient underwent repeat revascularization before treatment-pathway allocation or between treatment-pathway allocation and the TTT or ALBC procedure. Within 6 months postoperatively, six patients underwent unplanned lower-extremity revascularization because of ulcer progression or insufficient wound healing, including two patients in the TTT group and four patients in the ALBC group; these events were counted as URTOR and categorized as non-amputation salvage reoperation/reintervention. Later during follow-up, two additional patients underwent lower-extremity revascularization, both in the ALBC group; these events were recorded descriptively and were not included in the URTOR endpoint.

### Outcome evaluation

After stratification by Wagner grade, no significant differences were observed between the two groups regarding ulcer PAR at 3 months postoperatively. However, at 6 months, differences emerged in the incidence of URTOR, improvement in ΔABI, and time to infection clearance. Among patients who experienced URTOR, the composition of first events differed between treatment groups and wound grades (**Supplementary Table 2**).

In the Wagner grade 3 subgroup, no significant differences in ulcer PAR were observed at 3 months (P = 0.197) or 6 months (P = 0.336). Nonetheless, the TTT group experienced a significantly lower incidence of URTOR within 6 months (P = 0.012) and a pronounced improvement in ΔABI (P < 0.001) compared to the ALBC group. In contrast, the ALBC group demonstrated a significantly shorter time to infection clearance (P < 0.001). (**Table 2A**). Analysis of URTOR event composition (**Supplementary Table 2**) revealed that the majority of events in both groups were unplanned debridement or drainage (71.43% *vs*. 60.00%), with unplanned fixation/device revisions and minor distal amputations below the ankle being rare.

**Table 2A:** Outcomes and postoperative recovery indices in patients with Wagner grade 3 diabetic foot ulcers (TTT *vs* ALBC).

Outcomes	TTT group (n=45)	ALBC group (n=52)	P value
3-month ulcer PAR, median (IQR)	1.00 (1.00,1.00)	1.00 (1.00,1.00)	0.197
6-month ulcer PAR, median (IQR)	1.00 (1.00,1.00)	1.00 (1.00,1.00)	0.336
URTOR, n (%)	7 (15.56)	20 (38.46)	0.012
ΔABI, median (IQR)	0.11 (0.09,0.13)	0.04 (-0.03,0.08)	<0.001
Infection clearance time (days), median (IQR)	30.50 (26.25,37.75)	24.50 (22.00,27.00)	<0.001
Postoperative LOS (days), median (IQR)	12.00 (7.0,18.0)	12.00 (7.0,16.0)	0.836

PAR, percentage area reduction; URTOR, unplanned return to the operating room; ABI, ankle–brachial index; LOS, length of hospital stay; IQR, interquartile range. PAR values are expressed as proportions. Continuous variables are presented as mean ± SD or median (IQR), as appropriate, and categorical variables are presented as n (%).

**Table 2B:** Outcomes and postoperative recovery indices in patients with Wagner grade 4 diabetic foot ulcers (TTT *vs* ALBC).

Outcomes	TTT group (n=24)	ALBC group (n=17)	P value
3-month ulcer PAR, median (IQR)	0.837 (0.7,0.9)	0.781 (0.4,0.9)	0.723
6-month ulcer PAR, median (IQR)	1.000 (1.0,1.0)	0.906 (0.7,1.0)	0.002
URTOR, n (%)	11 (45.83)	14 (82.35)	0.018
ΔABI, median (IQR)	0.075 (0.1,0.1)	0.045 (0.0,0.1)	0.010
Infection clearance time (days), median (IQR)	35.0 (26.5,40.5)	26.0 (22.0,29.0)	<0.001
Postoperative LOS (days), median (IQR)	18.00 (11.3,39.3)	19.00 (10.0,30.5)	0.662

PAR, percentage area reduction; URTOR, unplanned return to the operating room; ABI, ankle–brachial index; LOS, length of hospital stay; IQR, interquartile range. PAR values are expressed as proportions. Continuous variables are presented as mean ± SD or median (IQR), as appropriate, and categorical variables are presented as n (%).

For the Wagner grade 4 subgroup, two patients in the TTT group developed progressive ulcer enlargement at 2 and 3 months postoperatively, respectively, and further received major amputation. Following the prespecified outcome definitions, these cases were classified as early treatment failures and were therefore excluded from subsequent analyses of ulcer PAR and ΔABI. Although there was no significant between-group difference in ulcer PAR at 3 months (P = 0.723), at 6 months, ulcer PAR was significantly higher in the TTT group relative to the ALBC group (P = 0.002). Similar to the findings in the Wagner grade 3 subgroup, the TTT group had a lower incidence of URTOR within 6 months (P = 0.018) and a greater improvement in ΔABI (P = 0.010). In contrast, the ALBC group demonstrated a significantly shorter time to infection clearance (P < 0.001), while LOS did not differ significantly between groups (P = 0.662) (**Table 2B**). Analysis of URTOR event composition (**Supplementary Table 2**) indicated that the TTT group had a higher proportion of minor distal amputations below the ankle (45.45%), while the ALBC group had a greater contribution from non-amputation salvage reoperation/reintervention (42.86%), with fixation/device revisions being uncommon across all groups.

**Figure 5:**
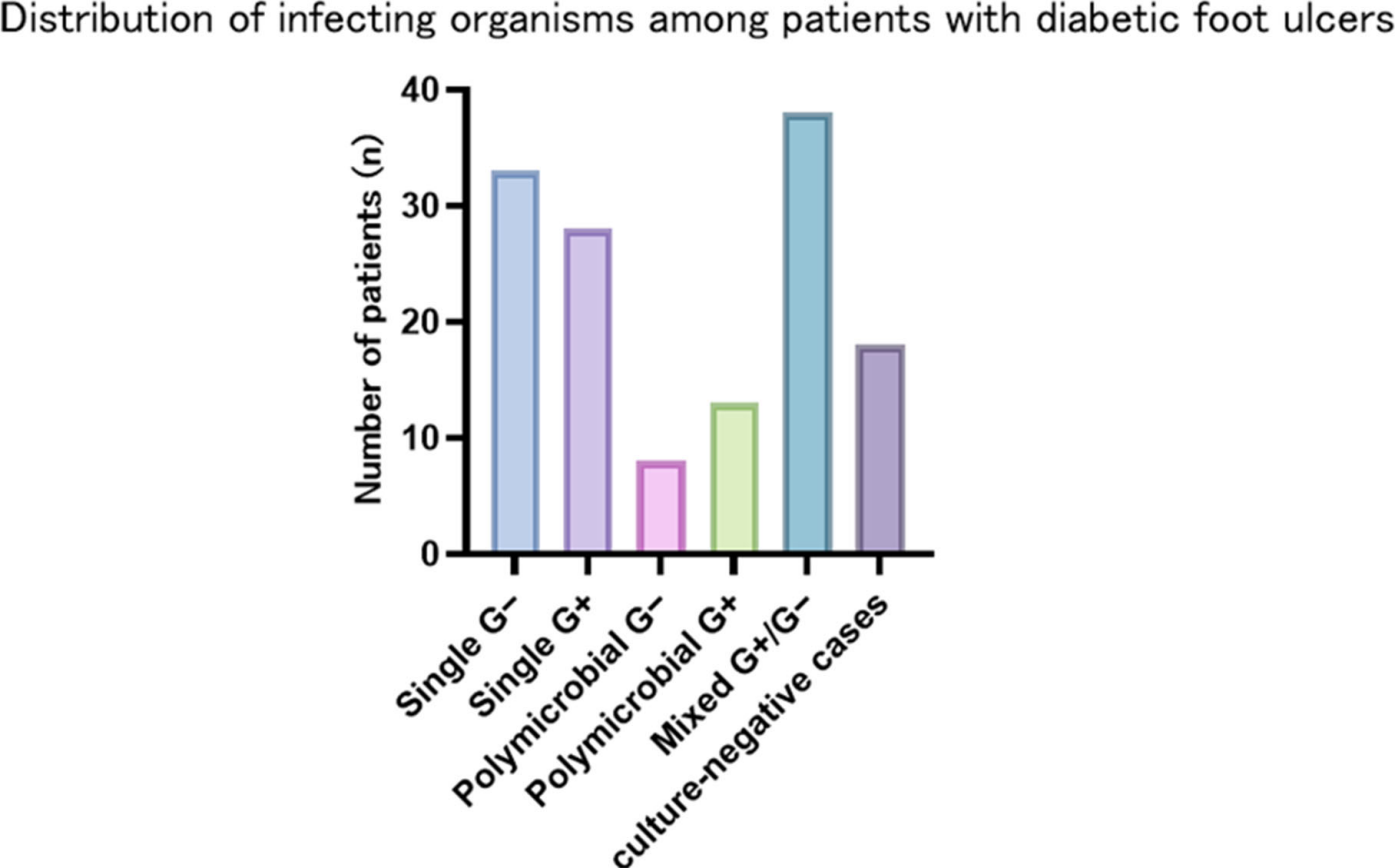
Kaplan–Meier curves for major amputation-free survival by treatment group.

Kaplan–Meier analysis showed that the mean major amputation-free survival time was numerically longer in the TTT group than in the ALBC group (23.789 months, 95% CI 22.634–24.943 *vs*. 18.362 months, 95% CI 17.753–18.971). However, the difference in major amputation-free survival between the two groups was not statistically significant (log-rank test, χ² = 0.239, P = 0.625) (**Figure 5**).

Because postoperative ABI may be influenced by baseline perfusion status and other clinical factors, a multivariable linear regression analysis was performed using postoperative 3-month ABI as the dependent variable. Multivariable linear regression analysis showed that the overall model was significant (R² = 0.912, adjusted R² = 0.909, F = 267.391, P < 0.001; **Table 3**). The treatment group was significantly associated with postoperative 3-month ABI (B = 0.075, SE = 0.011, standardized β = 0.215, P < 0.001). Preoperative ABI was also significantly associated with postoperative 3-month ABI (B = 1.060, SE = 0.032, standardized β = 0.959, P < 0.001). Osteomyelitis was positively associated with postoperative 3-month ABI (B = 0.046, SE = 0.015, standardized β = 0.116, P = 0.003), whereas abscess or deep soft tissue infection was negatively associated with postoperative 3-month ABI (B = -0.039, SE = 0.014, standardized β = -0.110, P = 0.006). Wagner grade was not significantly associated with postoperative 3-month ABI (P = 0.730)(**Table 3**).

**Table 3:** Multivariable linear regression analysis for postoperative 3-month ABI.

Variables	B	SE	Standardized β	t	P value
Wagner grade	-0.005	0.013	-0.012	-0.346	0.730
Treatment group(TTT *vs* ALBC)	0.075	0.011	0.215	6.850	<0.001
Osteomyelitis	0.046	0.015	0.116	3.055	0.003
Abscess or deep soft tissue infection	-0.039	0.014	-0.110	-2.823	0.006
Preoperative ABI	1.060	0.032	0.959	32.907	<0.001

Model summary: R = 0.955; R² = 0.912; adjusted R² = 0.909; F = 267.391; P < 0.001; Durbin–Watson = 2.093; n = 135.

SE, standard error.

In the multivariable logistic regression analysis for URTOR events, treatment group was significantly associated with URTOR (OR = 0.208, 95% CI 0.074–0.582, P = 0.003; **Table 4**). Preoperative ABI, calculated as preoperative ABI × 10, was also significantly associated with URTOR (OR = 0.582, 95% CI 0.422–0.802, P = 0.001), indicating the change in odds for each 0.1-unit increase in preoperative ABI. Wagner grade (OR = 2.745, 95% CI 0.927–8.129, P = 0.068), osteomyelitis (OR = 0.989, 95% CI 0.301–3.253, P = 0.986), and abscess or deep soft tissue infection (OR = 1.501, 95% CI 0.501–4.497, P = 0.468) were not significantly associated with URTOR in this model (**Table 4**).

**Table 4:** Multivariable logistic regression analysis for URTOR events.

Variables	B	SE	Wald	df	p value	OR	95% CI OR
Wagner grade	1.010	0.554	3.325	1	0.068	2.745	0.927-8.129
Treatment group(TTT *vs* ALBC)	-1.570	0.525	8.934	1	0.003	0.208	0.074-0.582
Osteomyelitis	-0.011	0.607	0.000	1	0.986	0.989	0.301-3.253
Abscess or deep soft tissue infection	0.406	0.560	0.526	1	0.468	1.501	0.501-4.497
Preoperative ABI	-0.541	0.164	10.928	1	0.001	0.582	0.422-0.802

ABI pre was calculated as preoperative ABI×10, so the OR represents the change in odds of URTOR for each 0.1-unit increase in preoperative ABI.

**Figure 6:**
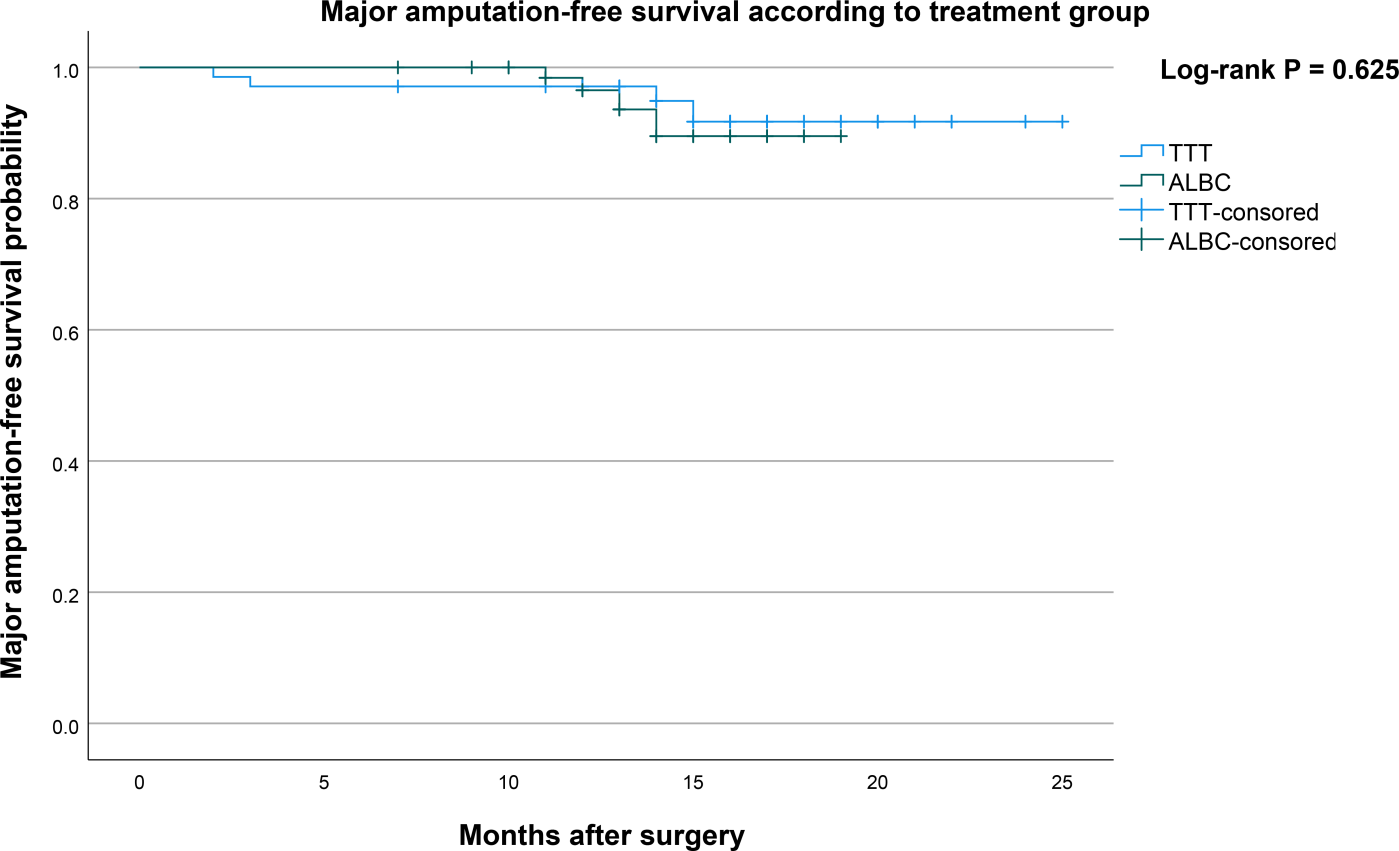
Distribution of microbial profiles in patients with diabetic foot ulcers.

**Figure 7:**
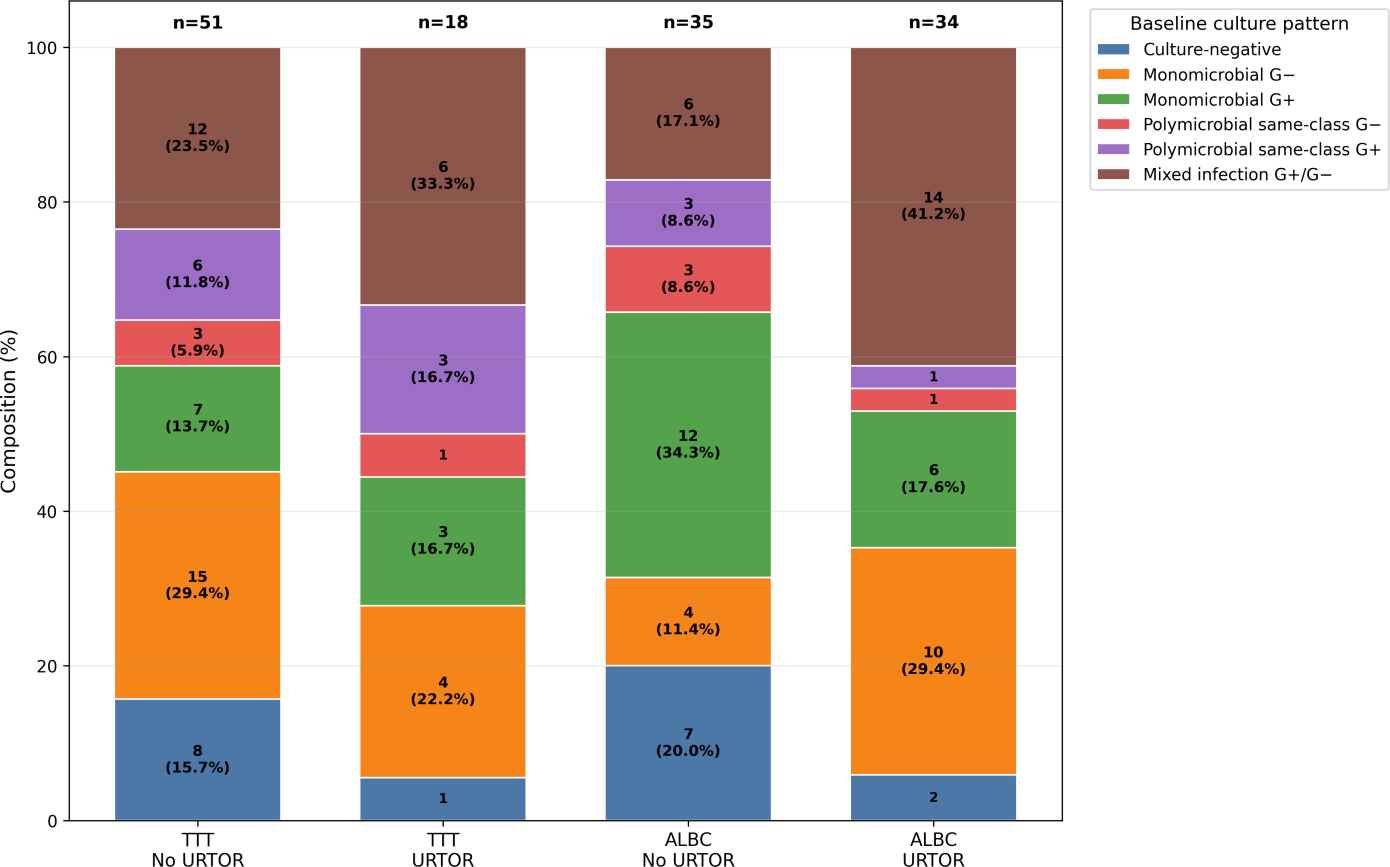
Baseline microbiological profiles stratified by treatment group and URTOR occurrence.

To further characterize the infection spectrum within the study cohort, we conducted a descriptive analysis of wound culture profiles in the overall population and according to treatment group and URTOR occurrence. In the overall cohort (**Figure 6**), mixed Gram-positive/Gram-negative infection was the most frequent pattern, followed by monomicrobial Gram-negative and monomicrobial Gram-positive infection, whereas polymicrobial same-class infections and culture-negative cases were less common. When stratified by treatment group and URTOR occurrence (**Figure 7**), the distribution of baseline microbiological profiles varied across subgroups. In the TTT group, monomicrobial Gram-negative infection was the most frequent pattern among patients without URTOR (15/51, 29.4%), whereas mixed Gram-positive/Gram-negative infection was the most frequent pattern among those with URTOR (6/18, 33.3%). In the ALBC group, monomicrobial Gram-positive infection was the most frequent pattern among patients without URTOR (12/35, 34.3%), while mixed Gram-positive/Gram-negative infection was the most frequent pattern among those with URTOR (14/34, 41.2%). In addition, monomicrobial Gram-negative infection accounted for 29.4% (10/34) of cases in the ALBC subgroup with URTOR.

### Typical cases

#### Clinical case 1

**Figure 8:**
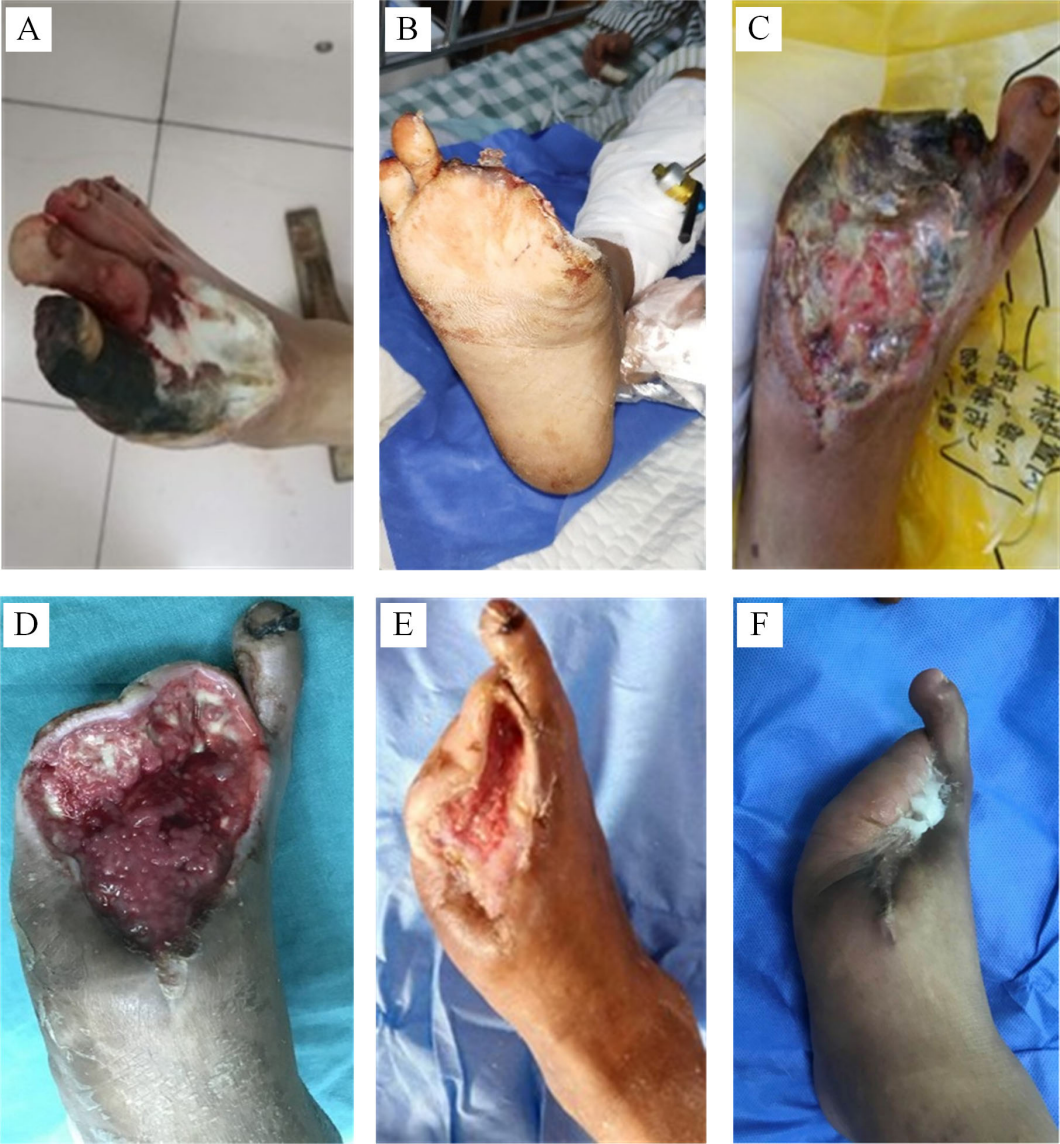
Treatment course of a 46-year-old male patient with Wagner grade IV DFU treated with TTT. **(A)** Preoperative status before TTT. **(B)** Status after extensive debridement and TTT. **(C)** Two weeks after TTT, prior to the second debridement. **(D)** Two weeks after second-stage debridement. **(E)** Five weeks after second-stage debridement. **(F)** Ten weeks after second-stage debridement.

A 46-year-old man was admitted with a Wagner grade IV diabetic foot ulcer. Preoperatively, gangrene was present in the right hallux, and the second, third, and fourth toes exhibited progressive ischemic necrosis. Initial limited debridement was performed to remove superficial nonviable tissue. Subsequent reassessment demonstrated clearly demarcated progression of ischemic necrosis. The patient underwent extensive debridement combined with TTT, resulting in amputation of the first, second, and third toes. At 2 weeks postoperatively, residual necrotic areas became further demarcated and a second debridement was performed. At 5 weeks following the second procedure, progressive granulation tissue formation and marginal re-epithelialization were observed without evidence of recurrent infection or new necrosis. Complete wound closure was achieved at 10 weeks after the second-stage debridement (**Figure 8**).

**Figure 9:**
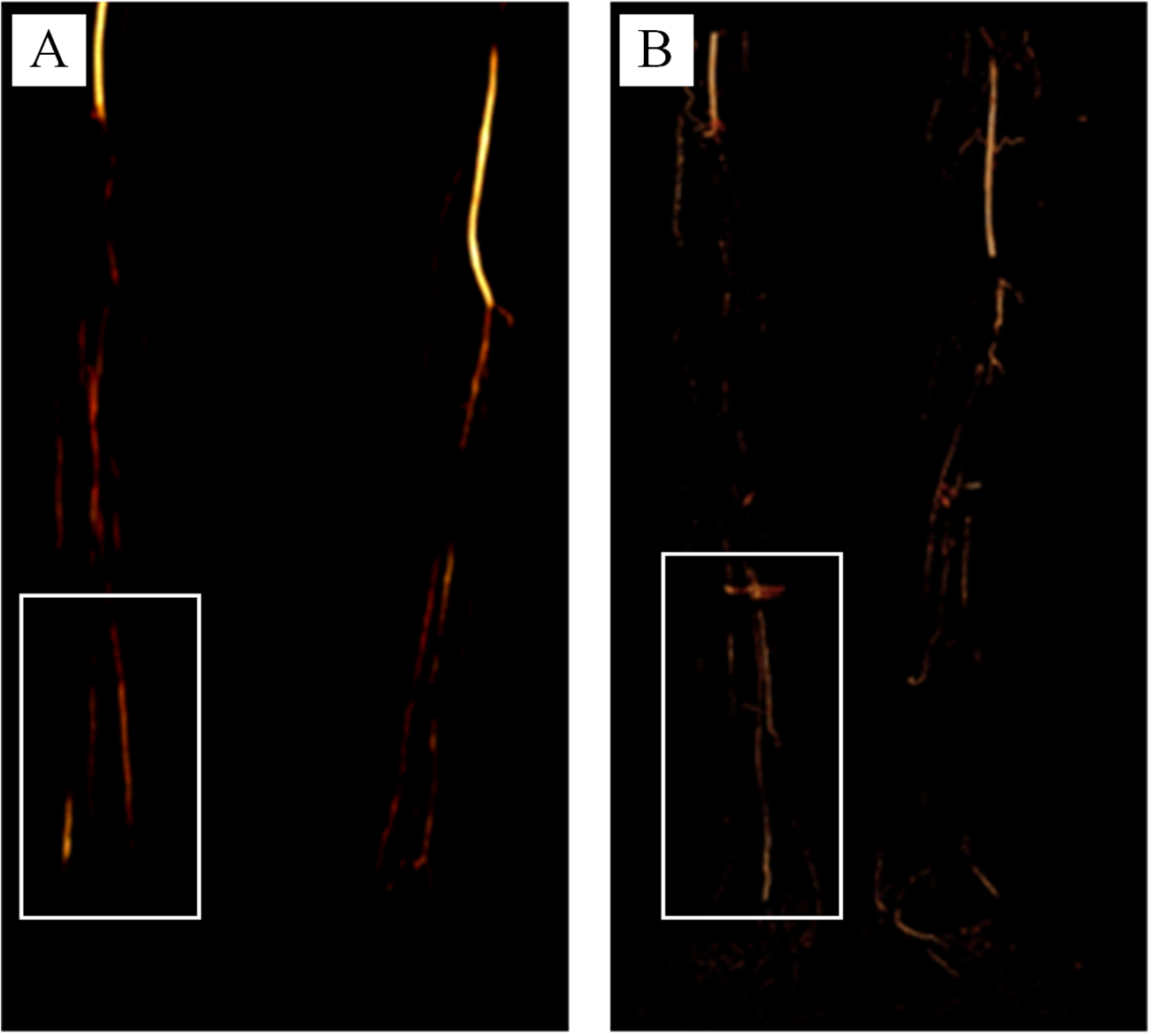
Three-dimensional CTA reconstruction in the same 46-year-old male patient with Wagner grade IV DFU (see **Figure 8**). **(A)** Preoperative CTA. **(B)** CTA at 2 months postoperatively.

Preoperative CTA revealed right popliteal artery stenosis. Postoperative CTA also showed right popliteal artery stenosis; however, development of collateral circulation was observed, indicating improved distal perfusion of the affected limb (**Figure 9**).

#### Clinical case 2

**Figure 10:**
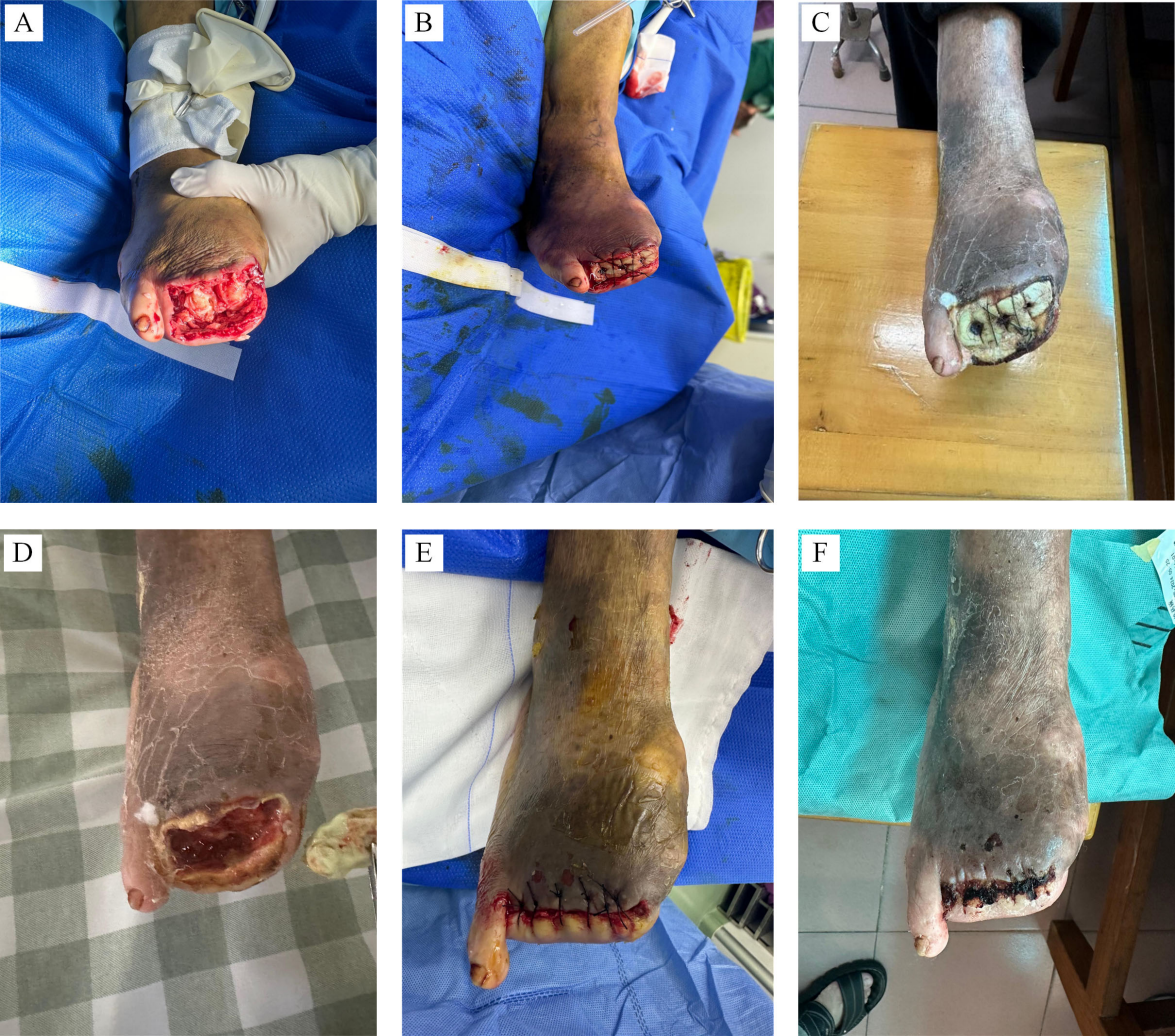
A representative case of a 62-year-old male patient with a Wagner grade IV refractory DFU treated with ALBC, illustrating the course of wound evolution. **(A)** Foot appearance after initial wound management. **(B)** After extensive debridement with local implantation of ALBC. **(C)** One week after ALBC implantation. **(D)** Three weeks after ALBC implantation, after bone cement removal. **(E)** Three weeks after ALBC implantation, after second-stage suturing. **(F)** Two weeks after second-stage suturing.

A 62-year-old man was diagnosed with a Wagner grade IV diabetic foot ulcer characterized by extensive local infection and tissue necrosis. Initial wound management consisted of limited debridement to remove superficial and visibly nonviable tissue. Follow-up evaluation revealed persistent infection with sinus tract formation, necessitating extensive debridement of the infected forefoot. After debridement, antibiotic-loaded bone cement (ALBC) implantation and vacuum sealing drainage (VSD) were applied. VSD was removed at 1 week postoperatively. Bone cement was removed at 3 weeks, at which time granulation tissue was observed at the wound base. Persistent wound exudation subsequently required repeat debridement, followed by secondary wound closure. Complete wound healing was achieved 2 weeks after secondary suturing, and the patient regained independent ambulation with an orthotic insole (**Figure 10**).

## Discussion

This study conducted a retrospective analysis of patients with Wagner grade 3–4 DFUs complicated by coexisting infection and perfusion impairment, comparing the clinical outcomes of two limb-salvage strategies targeting different pathological processes: ALBC and TTT. Notably, there was no significant difference between the two strategies in 3-month ulcer PAR in either subgroup following stratification based on the Wagner grade (Wagner 3: P = 0.197; Wagner 4: P = 0.723).At 6 months, ulcer healing rates remained comparable in Wagner grade 3 patients (P = 0.336), whereas TTT demonstrated a significantly higher healing rate in Wagner grade 4 patients (P = 0.002). Kaplan–Meier analysis demonstrated no significant difference in major amputation-free survival between the two treatment groups, although the TTT group showed a numerically longer mean amputation-free survival time. Given the low number of major amputation events in this cohort, this finding should be interpreted cautiously. Further stratified analysis revealed that the ALBC group had a significant advantage in early infection control (P < 0.001). In contrast, the TTT group, particularly among patients with Wagner grade 4 ulcers, exhibited a greater long-term ulcer PAR (P = 0.002) and a lower burden of URTOR (P = 0.018). These findings suggest that ALBC and TTT are both feasible limb-salvage options for complex DFUs, but their relative advantages differ based on the dominant pathological constraint. An important conceptual consideration is that ALBC and TTT target distinct but often coexisting pathological processes, namely infection and impaired perfusion. In clinical practice, combined or sequential approaches may be considered in selected cases. However, in the present study, patients were allocated to a single predefined pathway based on the dominant pathological process at the multidisciplinary reassessment time point. This design was intended to allow a clearer comparison of the primary therapeutic emphasis of each strategy, rather than to reflect all possible treatment combinations. The supplementary baseline data demonstrate that patients in the TTT group had a greater perfusion-related burden, whereas those in the ALBC group had a greater infection- and dead-space-related burden. These findings support the interpretation that treatment allocation reflected the predominant clinical constraint rather than arbitrary assignment. Therefore, the present study should be interpreted as a real-world comparison of two mechanistically distinct but clinically complementary, indication-driven limb-salvage strategies rather than as a direct head-to-head efficacy comparison between TTT and ALBC. Nevertheless, the absence of a fully validated quantitative scoring system to define the relative contribution of infection versus ischemia remains a limitation, and future studies should explore stratified or combined treatment strategies.

ALBC, as an efficient and stable antibiotic carrier, is primarily used as an adjunctive strategy for local infection control and dead-space management in clinical practice (23, 24). Although ALBC is intended to support local infection control, it does not directly enhance wound perfusion, which may result in wound stagnation due to persistent ischemia, consequently increasing the incidence of unplanned reoperations. Importantly, ALBC demonstrated a significantly shorter infection clearance time across both Wagner subgroups, with a difference of approximately 8–10 days. In the context of diabetic foot infections, where rapid progression can lead to systemic deterioration and limb loss, earlier infection control has clear clinical relevance (25). This finding supports the use of ALBC when infection control and dead-space management are the most urgent priorities in the early phase of treatment.

In contrast, TTT is designed to improve local perfusion through distraction-related vascular responses (26), providing a more favorable environment for wound repair (27). This improvement may clarify why TTT demonstrates a significant long-term healing advantage, especially in the treatment of more challenging Wagner grade 4 ulcers. However, the indication and positioning of TTT should be interpreted within the vascular-anatomical context of this study. In our treatment algorithm, perfusion impairment was evaluated using preoperative ABI and CTA-based assessment of distal arterial runoff. TTT was not intended to replace conventional revascularization in patients with correctable proximal arterial occlusion, nor was it used as a no-option ischemia therapy. Patients with superficial femoral artery or popliteal artery occlusion, or without effective distal arterial runoff, were not allocated to the TTT or ALBC pathway because they required priority vascular or interventional assessment for possible revascularization. Therefore, the present findings apply primarily to selected patients with severe DFUs who had preserved proximal arterial patency and residual distal runoff after standardized initial management. Within this context, TTT may serve as a perfusion-oriented adjunctive strategy to improve wound-healing conditions, rather than as an alternative to anatomically indicated revascularization. The observation that additional revascularization was still performed during follow-up when clinically indicated further underscores that TTT and ALBC were embedded within, rather than separated from, multidisciplinary limb-salvage care.

Multiple clinical studies have demonstrated that the perfusion status of the lower extremities is a crucial predictor of healing in diabetic foot ulcers (28, 29). The International Working Group on the Diabetic Foot guidelines emphasize that peripheral arterial disease (PAD) and perfusion evaluation are essential components for assessing prognosis and making treatment decisions (30). A systematic review demonstrated a significant association between insufficient peripheral blood flow and wound healing ability, particularly in patients with PAD, wherein inadequate perfusion directly impairs the wound healing process (31). We hypothesize that successful tissue repair relies on adequate improvement in perfusion (32). The conclusions of Wang et al. further confirmed the significant predictive advantage of perfusion improvement for healing (DOR = 15.81), with wound healing rates significantly reduced when TcPO_2_ < 30 mmHg (33). This study evaluates the improvement in macroscopic perfusion through ΔABI. The increase in ΔABI observed after TTT reflects improvement in macroscopic perfusion. Previous experimental and clinical studies suggest that TTT may promote angiogenesis and improve the local vascular environment, potentially contributing to enhanced tissue perfusion; however, whether this translates into meaningful enhancement of microcirculation cannot be directly determined without dedicated microcirculatory measurements such as TcPO_2_ or skin perfusion pressure (26, 34). For patients with Wagner grade 3 ulcers who have relatively good baseline perfusion, both treatment strategies appear to create adequate conditions for wound healing.

A perfusion-oriented treatment approach may be associated with fewer unplanned reoperations in clinical practice (28). Existing clinical evidence substantiates this rationale, as TTT not only facilitates the healing of refractory DFUs but also significantly decreases ulcer recurrence and the necessity for secondary interventions (17, 35). This indicates that patients are less likely to require further surgical interventions as a result of recurrence or progression (6). The data from this study support this finding, indicating that the TTT group experienced a lower URTOR rate in both Wagner grade 3 and 4 patients (both P < 0.05), as well as fewer major amputations in Wagner grade 4 patients. Notably, the very high URTOR rate observed in the Wagner grade 4 ALBC subgroup should be interpreted in the context of greater baseline infection severity and anatomical complexity in this group, rather than as a direct indication of treatment failure. This reduction in URTOR events is crucial for enhancing patient quality of life and conserving medical resources. Additionally, a study utilizing the California inpatient database demonstrated that the widespread use of revascularization treatment is closely associated with a decreased risk of amputation in PAD patients (36). Additionally, multi-center data have confirmed that PAD is an independent predictor of DFU amputation after controlling for infection and ulcer grade factors (OR = 1.554, P = 0.036), further supporting the significance of ischemia-related factors in amputation outcomes (37). However, the limited sample size in this study, particularly concerning major amputation as the outcome event, may result in insufficient statistical power to detect a significant correlation between perfusion deficiency indicators and major amputation events. This outcome likely reflects a lack of power rather than dismissing the potential role of perfusion status in amputation risk.

Furthermore, this study employed a single-center retrospective design, which is inherently subject to several limitations, including selection bias, information bias, and limited external validity. In addition, treatment allocation and clinical assessments were based on real-world multidisciplinary decision-making rather than predefined randomized protocols, which may introduce variability in clinical judgment and data recording. Treatment allocation was not randomized but was based on multidisciplinary clinical judgment and the dominant pathological process after standardized initial management. As shown in the supplementary baseline table, patients allocated to the TTT pathway had a greater perfusion-related burden, whereas those allocated to the ALBC pathway had a greater infection- and dead-space-related burden. Although this reflects real-world clinical decision-making and we performed multivariable adjustment for clinically relevant covariates, residual confounding and confounding by indication cannot be fully excluded. In addition, patients with major proximal arterial occlusion or absence of effective distal arterial runoff were excluded from the study population, as these patients required priority evaluation for possible revascularization. Therefore, the findings of this study may not be generalizable to patients with severe proximal arterial disease or no-option chronic limb-threatening ischemia, and should be interpreted within the context of patients with preserved proximal arterial patency and residual distal runoff. Future research could benefit from a multi-center prospective design to enhance the external validity of the conclusions. In addition, URTOR was analyzed as a composite endpoint that included heterogeneous events, such as repeat debridement, device-related revision, non-amputation salvage reoperation/reintervention, and minor distal amputation. Although we further categorized these events to improve interpretability, the sample size was insufficient to support robust statistical comparisons across individual URTOR subtypes. Therefore, the interpretation of URTOR should consider both event frequency and clinical heterogeneity. Moreover, although perfusion impairment was assessed using ABI and CTA-based distal runoff evaluation, ABI represents a macroscopic surrogate of perfusion and may be influenced by arterial calcification in patients with diabetes. Toe pressure, skin perfusion pressure, and TcPO_2_ were not routinely available in this retrospective cohort. Therefore, microcirculatory impairment and the degree of tissue-level ischemia could not be quantified in detail. Future prospective studies should incorporate standardized vascular-team assessment and objective microcirculatory parameters to better define which patients are most likely to benefit from perfusion-oriented procedures such as TTT. Furthermore, given the prolonged treatment process associated with DFU, future studies should extend the follow-up period to evaluate the impact of improvements in perfusion on long-term healing outcomes.

## Conclusion

In this retrospective cohort study of patients with Wagner grade 3–4 DFUs complicated by coexisting infection and perfusion impairment, the ALBC and TTT represented two distinct, indication-driven limb-salvage strategies. Short-term outcomes for ulcer healing and limb salvage were broadly comparable between the two strategies. However, ALBC was associated with faster infection clearance, suggesting that it may be more appropriate when infection control, osteomyelitis, abscess formation, or management of post-debridement dead space is the primary clinical concern. Conversely, TTT was associated with greater improvement in perfusion and a lower rate of URTOR, particularly in selected patients with preserved proximal arterial patency and residual distal runoff, where impaired distal perfusion may substantially hinder sustainable wound healing.

Overall, these findings support an individualized, pathology-oriented treatment framework following standardized initial management. Treatment selection should be guided by ulcer severity, infection burden, perfusion status, and vascular anatomy, with ALBC prioritized for infection control and dead-space management, and TTT considered for perfusion-oriented limb-salvage in selected patients with preserved proximal arterial patency and residual distal runoff. Rather than establishing superiority of one strategy over the other, this study highlights the importance of indication-driven treatment selection. Further prospective studies incorporating standardized microcirculatory assessment and validated decision algorithms are needed to translate this framework into actionable clinical guidance.

## Data Availability

The data analyzed in this study is subject to the following licenses/restrictions: The dataset used in this study was derived from hospital electronic medical records and contains potentially identifiable patient information. Therefore, it is not publicly available due to institutional and ethical restrictions. De-identified data may be made available from the corresponding author upon reasonable request and subject to approval by the relevant institutional authority and ethics committee. Requests to access these datasets should be directed to ML 1599782107@qq.com.

## Supplementary material

Supplementary Table 1 Baseline characteristics at treatment-pathway allocation after standardized initial management.

Supplementary Table 2 Breakdown of first URTOR events by treatment group and Wagner grade.
