# Supplementary material for: Clinical outcomes of tibial cortex transverse transport versus antibiotic-loaded bone cement for Wagner grade 3–4 diabetic foot ulcers: a real-world retrospective cohort study
Source: Front Endocrinol (Lausanne). 2026 Jun 3;17:1838948. doi: 10.3389/fendo.2026.1838948 (PMC13272054; doi:10.3389/fendo.2026.1838948)
Supplement: Supplementary Table 1 — Baseline characteristics at treatment-pathway allocation after standardized initial management. [file Table1.docx]

**Supplementary Table S1**. Baseline characteristics at treatment-pathway allocation after standardized initial management

| Characteristics | TTT group (n=69) | ALBC group (n=69) | P value |
| --- | --- | --- | --- |
| Wound grade, n (%) |  |  | 0.634 |
| W0 | 0 (0.0) | 0 (0.0) |  |
| W1 | 34 (49.28) | 35 (50.72) |  |
| W2 | 29 (42.03) | 25 (36.23) |  |
| W3 | 6 (8.70) | 9 (13.04) |  |
| Ischemia grade, n (%) |  |  | 0.059 |
| I0 | 5 (7.25) | 4 (5.80) |  |
| I1 | 10 (14.49) | 18 (26.09) |  |
| I2 | 36 (52.17) | 40 (57.97) |  |
| I3 | 18 (26.09) | 7 (10.14) |  |
| Distal arterial runoff on CTA, n (%) |  |  | ＜0.001 |
| 3-vessel runoff | 5 (7.25) | 19 (27.54) |  |
| 2-vessel runoff | 19 (27.54) | 30 (43.48) |  |
| 1-vessel runoff | 45 (65.22) | 20 (28.99) |  |
| Foot Infection grade, n (%) |  |  | ＜0.001 |
| Fi0 | 0 (0.0) | 0 (0.0) |  |
| Fi1 | 52 (75.36) | 19 (27.54) |  |
| Fi2 | 17 (24.64) | 49 (71.01) |  |
| Fi3 | 0 (0.0) | 1 (1.45) |  |
| Abscess or deep soft tissue infection, n (%) | 14 (20.29) | 43 (62.32) | ＜0.001 |
| Osteomyelitis, n (%) | 8 (11.59) | 29 (42.03) | ＜0.001 |
| Dead space management required, n (%) | 5 (7.25) | 33 (47.83) | ＜0.001 |

**Supplementary Table S2.** Breakdown of first URTOR events by treatment group and Wagner grade

| URTOR category | TTT,W3 (n=7) | ALBC,W3 (n=20) | TTT,W4 (n=11) | ALBC,W4 (n=14) |
| --- | --- | --- | --- | --- |
| fixation/device revision, n (%) | 1(14.29) | 1(5.00) | 0(0.00) | 0(0.00) |
| debridement/drainage, n (%) | 5(71.43) | 12(60.00) | 4(36.36) | 5(35.71) |
| Non-amputation salvage reoperation, n (%) | 0(0.00) | 5(25.00) | 2(18.18) | 6(42.86) |
| Minor distal amputation below the ankle, n (%) | 1(14.29) | 2(10.00) | 5(45.45) | 3(21.43) |
